# High-quality chromosome-level de novo assembly of the *Trifolium repens*

**DOI:** 10.1186/s12864-023-09437-8

**Published:** 2023-06-13

**Authors:** Hongjie Wang, Yongqiang Wu, Yong He, Guoyu Li, Lichao Ma, Shuo Li, Jianwei Huang, Guofeng Yang

**Affiliations:** 1 College of Grassland Science, Qingdao Agricultural University, Qingdao, 266109 China; 2 Key Laboratory of National Forestry and Grassland Administration On Grassland Resources and Ecology in the Yellow River Delta, Qingdao, 266109 China; 3 Berry Genomics Corporation, Beijing, China

**Keywords:** *Trifolium repens*, Genome assembly, PacBio HiFi, Genome annotation

## Abstract

**Background:**

White clover (*Trifolium repens L.*), an excellent perennial legume forage, is an allotetraploid native to southeastern Europe and southern Asia. It has high nutritional, ecological, genetic breeding, and medicinal values and exhibits excellent resistance to cold, drought, trample, and weed infestation. Thus, white clover is widely planted in Europe, America, and China; however, the lack of reference genome limits its breeding and cultivation. This study generated a white clover de novo genome assembly at the chromosomal level and annotated its components.

**Results:**

The PacBio third-generation Hi-Fi assembly and sequencing methods generated a 1096 Mb genome size of *T. repens*, with contigs of N50 = 14 Mb, scaffolds of N50 = 65 Mb, and BUSCO value of 98.5%. The newly assembled genome has better continuity and integrity than the previously reported white clover reference genome; thus provides important resources for the molecular breeding and evolution of white clover and other forage. Additionally, we annotated 90,128 high-confidence gene models from the genome. White clover was closely related to *Trifolium pratense* and *Trifolium medium* but distantly related to *Glycine max*, *Vigna radiata*, *Medicago truncatula*, and *Cicer arietinum*. The expansion, contraction, and GO functional enrichment analysis of the gene families showed that *T. repens* gene families were associated with biological processes, molecular function, cellular components, and environmental resistance, which explained its excellent agronomic traits.

**Conclusions:**

This study reports a high-quality de novo assembly of white clover genome obtained at the chromosomal level using PacBio Hi-Fi sequencing, a third-generation sequencing. The generated high-quality genome assembly of white clover provides a key basis for accelerating the research and molecular breeding of this important forage crop. The genome is also valuable for future studies on legume forage biology, evolution, and genome-wide mapping of quantitative trait loci associated with the relevant agronomic traits.

**Supplementary Information:**

The online version contains supplementary material available at 10.1186/s12864-023-09437-8.

## Background

**Fig. 1:**
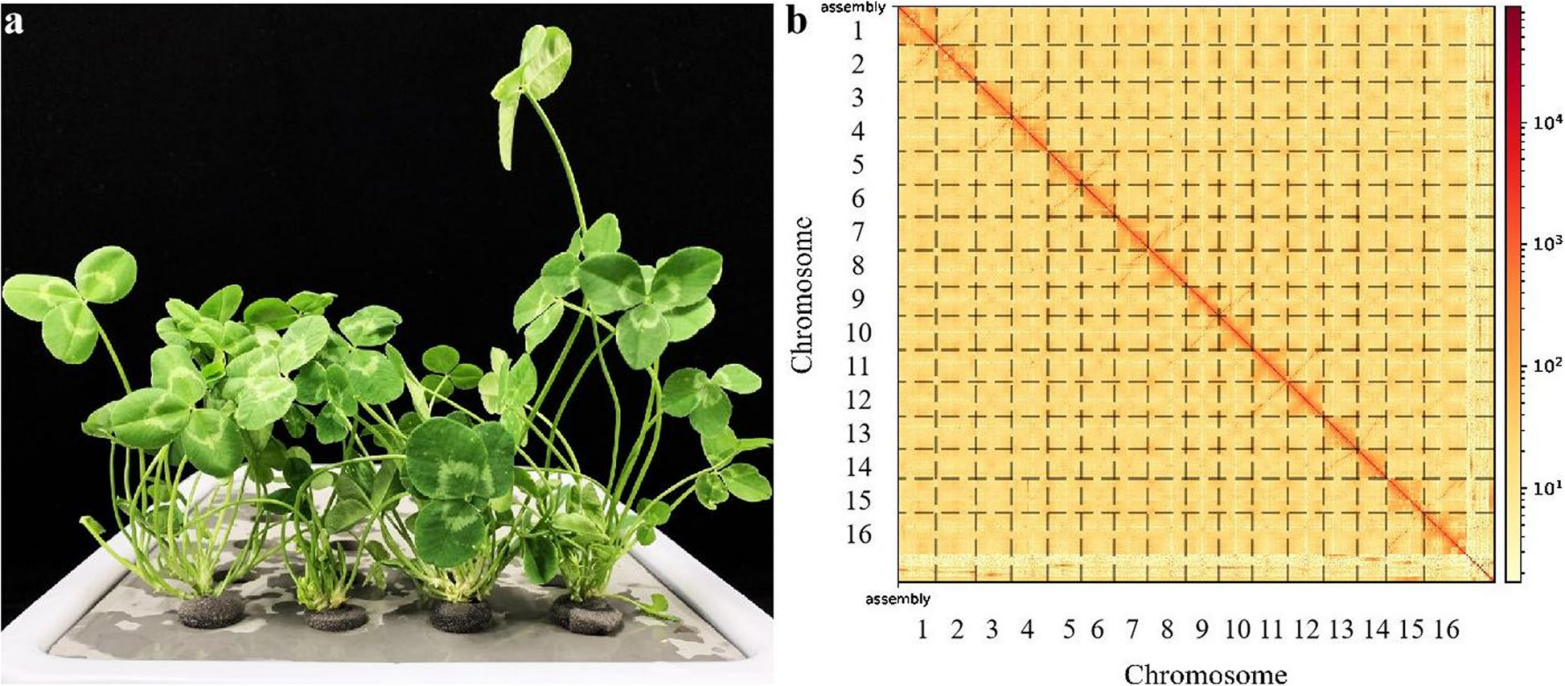
Plant morphology and Hi-C-assisted genome assembly of white clover (**a**) a Phenotype of the sequenced white clover plant. **b** Hi-C interaction heatmap showing 100-kb resolution super scaffolds

White clover (*Trifolium repens L.*) (Fig. 1a), an excellent perennial legume forage, is a heterotetraploid native to southeastern Europe and southern Asia. It is rich in diverse nutrients and mineral elements and has high nutritional, ecological, genetic breeding, and medicinal values [1–4]. The forage also has good palatability for herbivorous livestock, with high carbohydrate and protein content, and is used as ruminant feed in many parts of the world [5, 6]. Moreover, white clover is widely used as lawn ground cover for soil and water conservation due to its soil moisturization effect. White clover exhibits excellent growth when mixed with forages of the family Gramineae. It can play an integral role in intensive grazing systems regarding animal performance and herbage production, thus suggesting its important role in the stable development of the grassland ecosystem [6]. White clover has excellent resistance to cold, drought, trampling, and weed infestation, which is important for improving and breeding new varieties [7–10].

Compared with related species, such as alfalfa and soybean, the structural and genetic information of the white clover is limited, especially at the genomic level, greatly limiting its breeding and improvement [11–13]. Therefore, it is necessary to construct a high-quality white clover genome to accelerate its genetic research and fully use its genetic potential to breed excellent varieties [14].

Here, we use Illumina, PacBio, HiFi, and Hi-C (high-throughput chromatin conformation capture) technologies to generate a high-quality chromosome-level genome assembly of white clover [15, 16]. We annotated the components and functions of the white clover genome and conducted the genomic collinearity analysis between the white clover chromosome and the related species [17]. We also performed the protein family clustering analysis for the predicted genes. Furthermore, phylogenetic trees were constructed to estimate the differentiation time, and the contraction and expansion of gene families on each evolutionary branch were evaluated [18]. Forward selection gene analysis and genome-wide replication analysis were also performed. In summary, this study provides valuable genomic data for further studies and the breeding of white clover. The results of this study also provide a new research direction for analyzing the differentiation and evolution mechanism of white clover and the related species.

## Results

### Genome-survey, sequencing, and assembly

This study evaluated the size, repetitiveness, heterozygosity, and other genome parameters of the white clover. After quality control, Illumina sequencing yielded 59 Gb of data [19]. Blasting 10,000 randomly selected clean reads against the NT (Nucleotide Sequence Database) library revealed a 98.79% mapping. Moreover, K-mer analysis performed to estimate the complexity of the genome further predicted a genome size of 1075 Mb, with 68.80% repeat and 1.68% heterozygous sequences (Fig. S1). Traditional next-generation sequencing (NGS) data assembly methods were used to predict the genome size, while PacBio HiFi sequencing, a third-generation sequencing (TGS), was conducted for the white clover genome assembly [20]. High-quality Hi-Fi reads were obtained after parameter comparison of the output data. The Hi-Fi reads were 1.89 Mbp in size, with an N50 measure of 16.3kbp.

After eliminating heterozygous and redundant contigs, the assembled genome (1095 Mb) had 380 contigs, with an N50 of 14 Mbp and a maximum contig size of 53 Mbp. The average GC content of the assembled genome was 33.64% (Table 1).

**Table 1:** Summary statistic for the *Trifolium repens* genome

	Assembly	
Genome assembly	Estimated genome size	1075 Mb
	Total length of assembly	1096 Mb
	Number of contigs	380
	Contig N50	14 Mb
	Largest contig	53 Mb
	Number of scaffolds	202
	scaffold N50	65 Mb
	Chromosome coverage(%)	95.06%
	GC content of genome	33.64%
	Annotation	
		Total length
Transposable elements	Total Retrotransposon DNA Transposon	672 Mb(61.37%)
		448 Mb(40.91%)
		140 Mb(12.81%)
		Copies
Noncoding RNAs	rRNAs	10,984
	tRNAs	2,024
	miRNAs	662
	snRNAs	1352
Gene models	Number of genes	90,128
	Mean gene length	3,604 bp
	Mean coding sequence length	1,592 bp

To evaluate the quality and integrity of the assembly, we compared the sequencing data with the assembly results and found that the mapped ratio was 99.33%, with BUSCO (Benchmarking Universal Single-Copy Orthologs) assembly assessment integrity of 98.50% [21]. The BUSCO results of white clover assembly are shown in Table S1. These results indicate that the assembly had good integrity.

### Scaffold construction and curation

In this study, we used the Hi-C technology and generated 270 Gb of data, from which 180 Gb was used to construct chromosome-level super scaffolds with 160 times genome coverage. Subsequent analysis of the Hi-C library revealed a genome with a scaffold-Len of 1096 Mb and an N50 of 65 Mbp. Compared with the previously reported sequence data of white clover (scaffold N50 = 122 kb), the quality and integrity of the data obtained in this study were substantially higher [22].

Moreover, 95.06% of the contigs were attached to 16 chromosomes after the Hi-C-assisted assembly. The genetic material exchange was observed to be much stronger within than between chromosomes [23]. The statistical analysis results of chromosome sequence distribution are shown in Table S2. The heat map showing the genome interaction of the Hi-C-assisted assembly further verified the accuracy of the assembly results (Fig. 1b). Table 1 summarizes the assembly information. Thus, these results demonstrate the high accuracy of the Hi-C assembled genome.

### Genome annotation

The gene functions were inferred by analyzing the homology alignments and predicting the repetitive sequences. We constructed a repeat sequence library and annotated 2,023,411 repeat sequences. MITEs (miniature inverted-repeat transposable elements) and LTR (long terminal repeat) transposition components were identified by the structure prediction method, and these elements accounted for 61.37% and 37.75% of the total sequences, respectively. Copia and Gypsy accounted for 13.56% and 11.49% of LTR-retrotransposons, respectively. The results of the repetitive sequences are shown in Table S3.

Additional 4092 simple repeats were also found in the assembled genome, and we predicted 13 types of ncRNA, totaling 15,520 ncRNAs. After removing the gene models containing premature stop codons and frameshifts, we obtained 90,128 high-confidence gene models and 91,690 transcripts using RNA-seq and de novo prediction strategies. However, these gene models were unevenly distributed across the 16 chromosomes.

Each gene contained an average of one transcript, and the average lengths of white clover genes and transcripts were 3604 bp and 1697 bp, respectively. Moreover, each transcript contained an average of 5 exons, with average lengths of 341 bp. We also compared the white clover genome with its five closely related species, including *Medicago truncatula*, *Trifolium medium*, *Vigna radiata*, *Cicer arietinum*, and *Glycine max*. The results showed that *T. medium* (119,102) had the most genes, while *V. radiata* (29,006) and *Cicer arietinum* (28,772) had the least. The five species had similar average coding sequence (CDS) lengths except for *T. medium* (306) (Table 2)*.*

**Table 2:** The information of annotated gene models per species for all the species

Organism	Number of genes	Mean CDS length (bp)	Exons per transcript	Mean exon length (bp)	Mean intron length (bp)
*Vigna radiata*	29,006	1430	7.6	293	449
*Glycine max*	54,881	1391	8	295	413
*Trifolium medium*	119,102	306	1.4	219	172
*Cicer arietinum*	28,772	1393	7.7	291	418
*Medicago truncatula*	36,079	1428	6.9	324	393
*Trifolium repens*	90,128	1592	5	341	490

**Fig. 2:**
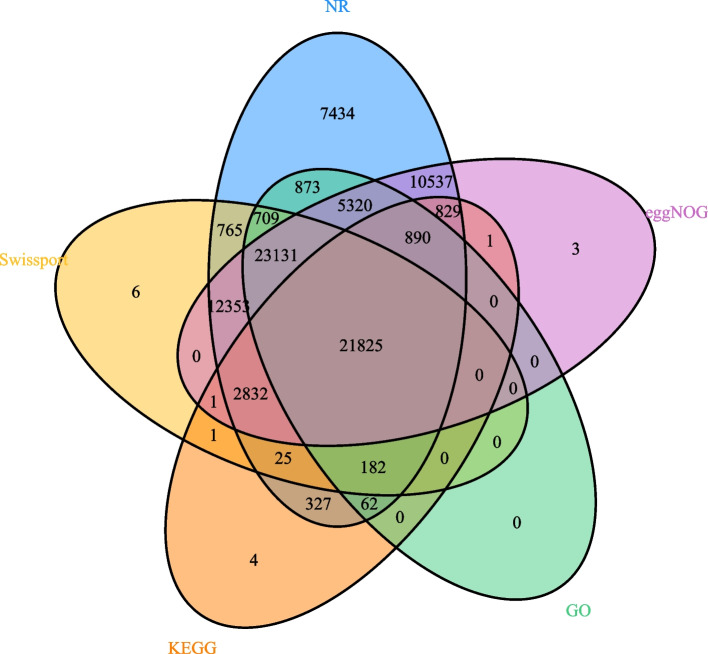
Venn analysis of five major databases (NR, Swiss-Prot, eggNOG, GO, KEGG) containing gene function annotation information

Using the NR, SwissProt, KEGG, GO, and eggNOG databases, we annotated and predicted the function and number of various genes [24]. We annotated 88,094, 61,830, 77,722, 52,992, and 26,979 genes using NR, Swiss-Prot, eggnog, GO, and KEGG databases, respectively. Furthermore, we conducted a Venn analysis by integrating the five databases, which revealed 21,825 common gene annotations (Table S4). Venn analysis of functional gene annotations is shown in Fig. 2.

### Gene family and evolution analysis

**Fig. 3:**
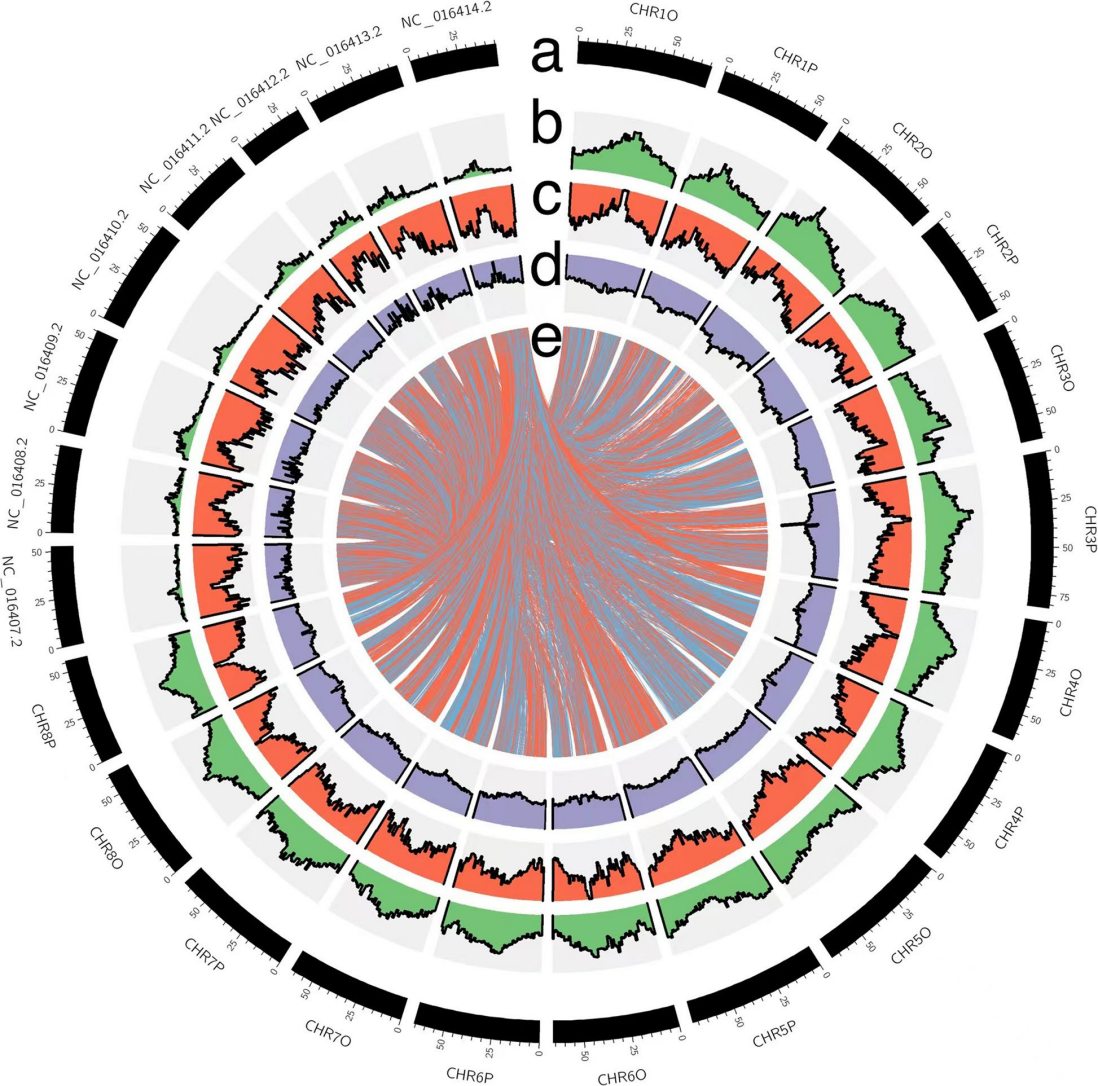
Features of *T. repens* and *M. truncatula* genome. **a** Length of each pseudochromosome (Mb). **b** Distribution of repetitive sequence. **c** Distribution of gene density. **d** Distribution of the GC content (**e**) *T. repens* and *M. truncatula* synteny analysis; the beginning of NC represents the chromosome of *M. truncatula*, while the beginning of CHR represents the chromosome of *T. repens*

Closely related species tend to have greater collinear fragments coverage and the collinear relationship between their genomes. Collinearity analysis suggested that the relationship between *T. repens* and *M. truncatula* is relatively close. Moreover, 16 chromosomes of *T. repens* and eight of *M. truncatula* had a good collinear relationship (Fig. 3), indicating their chromosomal conservation after species divergence [25].

**Fig. 4:**
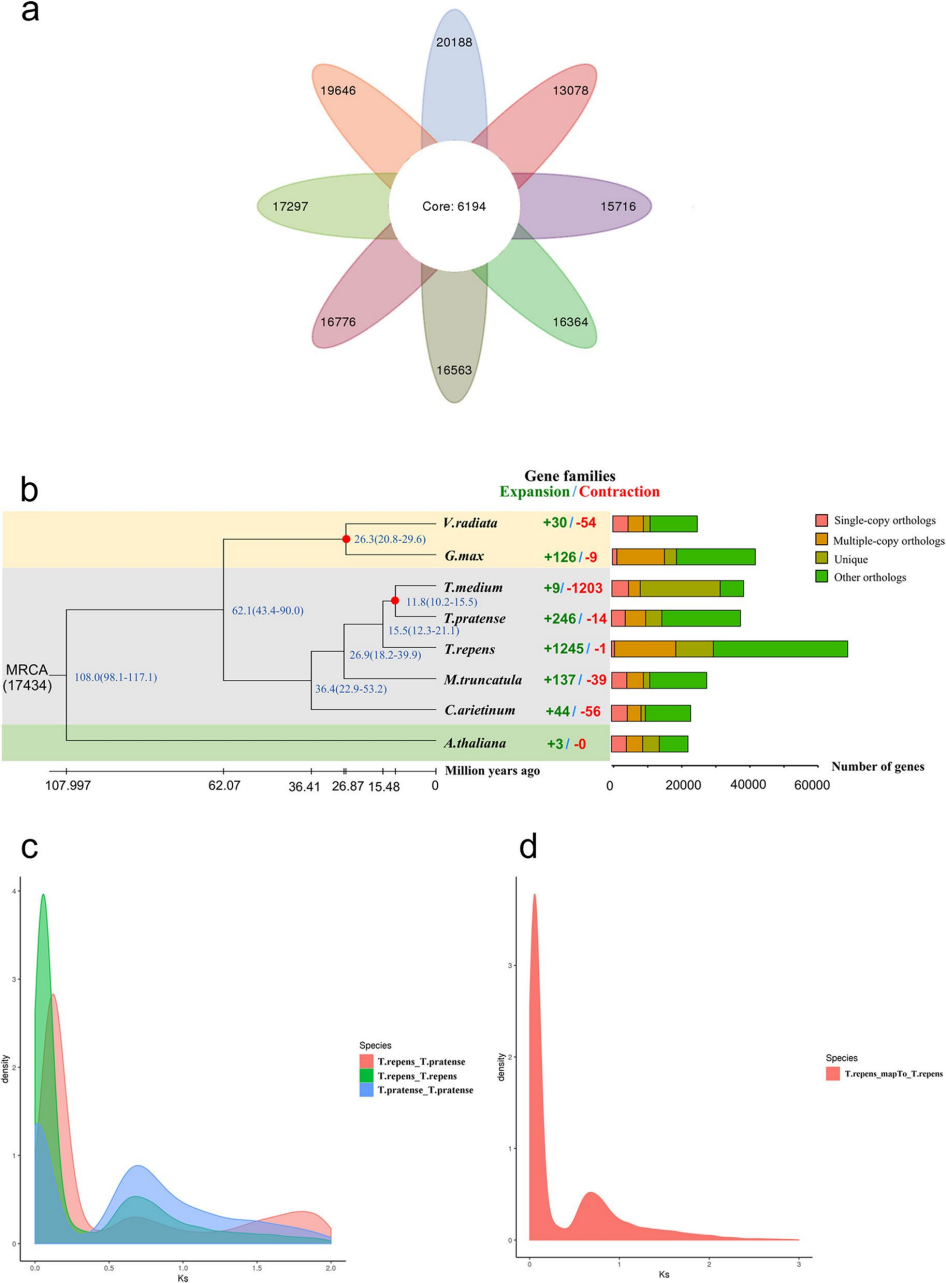
Gene family and phylogenetic tree analyses of white clover and other representative plant genomes. **a** Venn diagram of the number of shared gene families. **b** A phylogenetic tree based on shared single-copy gene families (left), gene family expansions and contractions among white clover and seven other species (middle), and Gene family clustering in white clover and seven other plant genomes (right). **c** Genome-wide replication Ks distribution map of white clover and its related species. **d** Genome-wide replication Ks analysis of white clover

The *T. repens* genome assembled in this study was compared with the genomes of seven other related species *G. max*, *V. radiata*, *M. truncatula*, *T. medium*, *C. arietinum*, *Arabidopsis thaliana*, and *T. pratense*. The OrthoMCL clustering analysis showed that 90,128 white clover genes clustered into 25,840 gene families. *Arabidopsis* had the most gene families (26,382), and *T. repens* shared 6,194 gene families with the seven related species (Fig. 4a). Cafe software was used to study the changes in gene families among the species at a family-wide *p*-value threshold of 0.05. The analysis showed that the red trifoliate significantly expanded 1,245 gene families but contracted one gene family during evolution (Fig. 4b) [26]. Distributions of the single-copy genes, multi-copy genes, endemic genes, and other types of genes per species are shown in Supplementary Figure S2.

GO functional enrichment analysis revealed the expansion of gene families related to protein phosphorylation, transmembrane transport, adenine nucleotide binding, and membrane composition. Furthermore, *T. repens* gene families were associated with biological processes, molecular function, cellular components, and environmental resistance, which could explain its excellent agronomic traits (Table S5). The positive selection analysis model was established with white clover as the foreground branch and other species as the background branch. Finally, three genes with significant positive selection were obtained.

We constructed a phylogenetic tree based on the results of protein family clustering and found that *T. repens* formed a monophyletic group with *V. radiata*, *G. max*, *T. pratense*, *T. medium*, *M. truncatula*, and *C. arietinum* [27]. White clover was most closely related to *T. pratense* and *T. medium*, with their estimated divergence time being 15.5 million years ago (Fig. 4b).

Whole genome duplication (WGD) events are important indices of plant evolution and are the driving force for plant adaptation to various environments [28]. Thus, WGD provides sufficient genetic material for expanding plant gene families or generating new genes. It also enhances the adaptability of plants to the environment and accelerates the evolution of plants by generating various genetic variations. To explore the evolutionary history of *T. repens*, we used the changes in the synonymous replacement rate of paralogous genes to measure gene duplication and loss in its genome. The resultant data suggested that the divergence of *T. repens* and *T. pratense* occurred after the WGD events. Both *T. repens* and *T. pratense* experienced a WGD event when the K_S_ value was 0.13 (Fig. 4c); however, an additional WGD event also occurred when the K_S_ value of *T. repens* was 0.6 (Fig. 4d).

## Discussion

Leguminous forages have excellent agronomic traits, and their genomic data are important for genetic analysis, breeding, and functional omics. White clover is a forage and lawn grass widely grown worldwide. Assembling white clover (*T. repens*) is challenging due to its large genome structure and highly homologous genomic sequences. However, this study assembled a high-quality tetraploid white clover genome using the latest third-generation Hi-Fi assembly and sequencing methods, providing a good reference for the research on other herbage of the Clover genus.

Compared with the second-generation sequencing technology, TGS technology overcomes some NGS shortcomings in genome assembly. TGS does not require polymerase chain reaction (PCR) amplification or long read length and has no guanine-cytosine (GC) preference, thus making genome assembly using PacBio Hi-Fi an effective assembly strategy [29, 30]. Compared with the previously published 841 Mb (N50 = 122 kb) genome assembly of white clover, the genome size in this study was 1,095 Mb (contig N50 = 14 Mbp), indicating a significantly improved quality. The average GC content of the assembled genome was 33.64% (Table 1), close to the previously assembled *Trifolium repens* genome (35%). Additionally, the number of newly assembled white clover reads was 400,467,170, and the mapping ratio was 99.33%, higher than that of the previously assembled genome (98%). Compared with the previously reported total BUSCO groups (1321), the assembly in this study had 2326 total BUSCO groups, and the Fragmented and Missing BUSCOs were smaller than the previous assembly. Moreover, complete Single-Copy BUSCOs (98.5%) were higher in the present than in the previous assembly (92%). Thus, the newly assembled white clover genome had better continuity and integrity than its previously reported reference genome [22].

Thus, our work has provided a chromosomal-level genome assembly using Hi-C-assisted genome assembly of white clover based on the whole-genome data. The technique utilizes the entire cell nucleus to fix and capture the mutual chromosomal sites [31, 32]. Hi-C uses high-throughput sequencing to determine the whole-genome spatial distribution of chromatin DNA through a high-resolution interaction map of chromatin regulatory elements obtained from the positional relationship [33, 34]. The published examples of higher plants assembled with Hi-C-assisted genomes include quinoa, barley, durian, and so on [32, 35, 36]. In this study, the assembly generated contains 202 scaffolds (~ 1096 Mb) spanning N50 = 65 Mb, with significantly improved quality. The assembled genome had higher coverage (95.06%) at the chromosomal level after high-throughput sequencing and Hi-C scaffolding.

We annotated 90,128 high-confidence gene models from the newly assembled genome. The published assembled genome annotated 68,558 genes, and the average CDS length was larger than the reported genome [22]. A high-quality reference genome of *T. repens* is important for understanding its evolution, origin, and domestication history. Therefore, this study provides important resources for molecular breeding and evolution analysis of white clover and other forages [37].

*T. occidentale *and *T. pallescens* are reportedly the progenitors of white clover, which originated about 15–28,000 years ago from multiple hybridization events during the last glaciation. Therefore, its evolutionary history is not well-understood. Genomic collinearity analysis showed that *T. repens* and *M. truncatula* exhibited close phylogenetic and genetic relationships. Moreover, phylogenetic analyses revealed that *T. repens* diverged after *V. radiata*, *G. max*, *M. truncatula*, and *C. arietinum* but before *T. medium* and *T. pratense * [38]. Thus, these species share the same ancestry with *T. repens*.

This study focused on comparing the genomes of white clover and related species at the genomic level. The structural genome characteristics, gene function, and evolutionary status of white clover were explained by the collinearity analysis between related species and intraspecies. Moreover, whole-genome replication events, phylogenetic tree construction, and differentiation time estimation, gene protein family clustering, contraction/expansion analysis, gene retention and loss, and forward selection gene analysis were also conducted. In summary, we decoded the complex white clover genome, revealed the events that have shaped the genome, and created foundations for further studies on legumes and complex genome assembly [20, 38]. The newly assembled genome is also valuable for future studies on white clover biology, evolution, and genome-wide mapping of quantitative trait loci associated with its agronomic traits.

Future research on this work will focus on the in-depth evaluation of specific traits of white clover, transcriptome sequencing, or large-scale population resequencing of specific tissue sites or growth and development periods. We will also consider using high-resolution single-cell technology to conduct single-cell transcriptome analysis of specific tissue sites in an attempt to solve the molecular mechanism of white clover resistance to various stresses. This will provide a valuable reference for further studies and utilization of white clover, an important forage resource.

## Conclusions

This study reported a high-quality de novo assembly for white clover obtained at the chromosomal level using PacBio third-generation Hi-Fi sequencing. The newly assembled genome has outstanding coverage and integrity; thus provides a key basis for accelerating the research and molecular breeding of this important forage crop. The genome is also valuable for future studies on white clover biology, evolution, and genome-wide mapping of quantitative trait loci associated with its agronomic traits.

### Experimental procedures

The *T. repens* (2n = 4x = 32) was planted in a light incubator at the Key Laboratory of National Forestry and Grassland Administration on Grassland Resources and Ecology in the Yellow River Delta. Thereafter, five-week-old leaf samples were sampled from each white clover into vacutainer tubes for genomic DNA extraction. The study complied with the ethical norms of Chinese and international regulations.

### DNA and RNA extraction

The *T. repens* (white clover Super Haifa) plants were grown in a phytotron chamber at 25 °C at the Qingdao Agricultural University in Shandong, China, under the photoperiod of 16/8 h, a light intensity of 400 W/m ^2^, and relative humidity (RH) of 70%. The leaf samples were collected and treated with liquid nitrogen for DNA extraction using the Tiangen DNA Secure Kit for Genome Sequencing (Beijing, China). Total RNA was extracted using an EASYspin Plus Polysaccharide Polyphenols/Complex Plant RNA Rapid Extraction Kit, following the manufacturer’s instructions.

### Survey analysis

The quality and quantity of DNA samples were controlled, and the qualified DNA samples were randomly broken into fragments by Covaris ultrasonic fragmentation instrument. Library preparation was conducted by terminal repair, a-tail addition, sequencing connector addition, purification, and PCR amplification. The libraries were then subjected to paired-end 150 (PE150) sequencing using Illumina NovaSeq [39–41]. The original image data file sequenced by the high-throughput sequencer was converted into the original sequence by base calling analysis [39]. To obtain clean reads, we filtered the off-plane reads to remove joints with low numbers, repeated and low-quality reads that would affect the comparison quality and subsequent analysis. We randomly selected and blasted 10,000 clean reads against the NCBI non-redundant nucleotide database (NT library) to check for possible external contamination [42].

K-mer analysis using Jellyfish software estimated the genome size, sample heterozygosity, and genome repeat sequence ratio (Table S6) [43]. The genome size of white clover was estimated using the following formula: G = Knum/Kdepth, where Knum is the number of k-mers, while Kdepth is the expected depth of k-mers.

### Genome assembly and quality evaluation

Minia was used for preliminary assembly with second-generation data (Table S6), and the assembly results were evaluated using the GC_depth analysis. DNA concentration and purity were measured by NanoDrop 2000 spectrophotometry. After sequencing with PacBio SMRT technology, a PCR-free SMRTbell library was constructed from a high-quality purified genome through repair and end-joining [44]. The library size was then determined by pulsed-field electrophoresis, and the acquired data were filtered and loaded onto smrtlink (Table S6) for CCS (Circular Consensus Sequencing) processing. The original PacBio Sequel data, the Polymerase Reads, were filtered to obtain subsequent available SubReads, which were then processed with smrtlink software for CCS to obtain high-quality HiFi Reads (Table S6). To obtain high-quality Hi-Fi Reads, we conducted CCS on the SubReads obtained above using parameters –min-passes = 3 –min-rq = 0.99.

Hifiasm software (Table S6) was used for assembly, and all-vs-all alignment was used to correct sequencing errors [20, 44, 45]. Overlap comparison was repeated after correction, and a phased string graph was constructed [41]. Finally, contigs were generated based on the overlapping graph, and the assembly contig sequence was further deheterozygosed through purge_dups(v1.2.3) (Table S6) [46, 47]. The assembled genome was compared with HiFi Reads using the software Minimap2 (Table S6), and then heterozygotic fragments were removed based on the coverage distribution and sequence score of the reads [48]. Pseudo contigs is removed from the genome by BWA (Burrows-Wheeler-Alignment Tool) (Table S6). After redundancy analysis, the genome sequence was compared with the second-generation data, and the GC-depth graph was generated. Contigs with average coverage depth less than 5X was removed. In addition, contigs with window GC content of 50%-53% were also removed, and the final assembly result was calculated.

The sequencing data was compared with the assembly results to evaluate the data recovery ratio and integrity assessment was conducted using BUSCO (Table S6) and the BUSCO Eudicots lineage dataset (eudicots_odb10) [21]. The genome assembly results were evaluated based on the proportion of matched read pairs and the distribution of inserted fragments. Tblastn (Table S6), Augustus, and Hmmer tools were used to evaluate the integrity of the single-copy orthologous genes [21]. Genome sequencing was performed by Berry Hekang (Beijing, China) using the third-generation PacBio Sequel II sequencing platform.

### Hi-C data analysis and chromosome construction

For DNA cross-linking, we soaked 100 mg of *T. repens* leaf tissues in paraformaldehyde (a cell cross-linking agent) for 15 min, after which glycine was added to terminate the chromatin cross-linking reaction. The treated tissues were collected, frozen in liquid nitrogen and ground for DNA extraction. Biotin-labeled oligonucleotide ends were added during the terminal repair, and the adjacent DNA fragments were linked with nucleic acid ligase. The protein was enzymatically cleaved at the junction point with protease, and the Covaris crusher was used to randomly break up 350 bp of DNA [35, 49]. Biotinylated DNA fragments were bound to avidin magnetic beads to create the whole library. After qualified library analysis, different libraries were pooled for Illumina PE150 sequencing according to the concentration and target requirements for machine data volume [19]. Thereafter, 10,000 pairs of sequencing reads were randomly selected from the Hi-C sequencing database and blasted against the NT library (Table S6). The top 10 matched species were sequenced and evaluated to determine whether there was bacterial contamination. The JUICER (Table S6) software was then employed to compare the Hi-C data with the draft genome [31, 45, 50]. We analyzed the Hi-C library results via 3D-DNA (Table S6) comparison to obtain valid Hi-C data and generate the chromosome-level scaffold of the white clover genome [31, 45]. After the Hi-C-assisted assembly was completed, the interchromosome and intra-chromosome exchanges were calculated to further verify the accuracy of the assembly results [19].

### Genome annotation

Repetitive sequences of the white clover genome were annotated using homology-based and ab initio search methods [51, 52]. Class II transposition factor mites and involuntary transposition factors less than 2 kb in length were searched in the genome using MITEs [53]. To obtain more reliable LTR-RT, we used an LTR retriever to analyze the process. We combined LTRharvest (-similar 90 -vic 10 -seed 20 -seqids yes -minlenltr 100 -maxlenltr 7000 -mintsd 4 -maxtsd 6 -motif TGCA -motifmis 1) with LTR Finder results to filter false-positive LTR-RT (Table S6) [54, 55]. Repetitive sequences of known species were searched in the RepBase library using RepeatMasker (http://www.girinst.org/server/RepBase/index.php) in combination with MITEs.lib library and Lcr.lib library. The combination library was then used as the database to shield the repetitive sequences of the genome using RepeatMasker (Table S6), which were re-identified using RepeatModeler (Table S6). The sequences classified as unknown by the RepeatModeler were compared with the transposable enzyme database using Blastx, and reclassified according to the transposable enzyme type.

The tRNA ab initio rRNA was predicted using tRNAscan-SE (Table S6) software [56], and the other types of ncRNA were searched using the Rfam database (ftp://ftp.ebi.ac.uk/pub/databases/Rfam/14.1/) [56–59]. The specific information of these RNA types was obtained through similarity comparison.

All repetitive regions except the tandem repeats were soft-masked for protein-coding gene annotation. The coding sequences of *M. truncatula* (GCF 000219495.3 MedtrA17 4.0), *T. medium* (GCA 003490085.1 ASM349008v1), *V. radiata* (GCF 000741045.1 *Vradiata* ver6), *C. arietinum* (GCF 000331145.1 ASM33114v1), and *G. max* (GCF 000004515.5 *Glycine max* v2.1) were downloaded. These coding sequences were then subjected to blast (Table S6) searches against the white clover genome, and the homologs containing premature stop codons and frameshifts were discarded [42]. GeMoMa-1.6.1 (Table S6) was used to compare the protein sequence of the related species with the assembled genome to predict their gene structure. Meanwhile, the boundary information of exon and intron was obtained by comparing RNA data with the assembly results. High-quality full-length transcripts were established through the iso-seq standardization process in SMRT analysis software and used to predict the open reading frames (ORFs) via PASA v2.0.1 (Table S6). The protein sequences were filtered to 100AA ~ 1000AA and a CDS number of ≥ 2. A gene that matched the full length of the reference protein sequence was obtained, and the cDNA sequence of the gene was used as the training set. Augustus, SNAP, GlimmerHMM, and GeneMark-ESSuite (Table S6) were used to predict the gene structure [60]. The training set was used for parameter training, and the intron hints indicated that the RNA-Seq reads and scaffolds were comparable. The compared reads were then combined with intron hints for gene structure prediction. The predictions obtained using these packages were combined using EVM (Table S6), after which 36,511 genes were retrieved and functionally annotated by blast searches against NR (ftp://ftp.ncbi.nlm.nih.gov/blast/db/), Swiss-Prot (ftp://ftp.ebi.ac.uk/pub/databases/uniprot/knowledgebase/uniprot_sprot.fasta.gz), eggNOG, GO (http://geneontology.org/), and KEGG (http://www.genome.jp/kegg/) databases. Venn analysis of these databases was then performed to obtain more accurate gene functional annotation information [61].

### Genome comparative analysis

We conducted genome collinearity analysis of the white clover and its relatives using the Mummer software (parameters: nucmer -g 1000 -c 90 -l 200) and Lastz (Table S6) [62, 63]. To determine the similarity between sequences, we used OrthoMCL (Table S6) clustering analysis to perform all-VS-All BLAST alignment on gene protein-coding sequences of all selected species (e-value = 1e-5 by default) [64]. Markov clustering algorithm was used for clustering analysis (expansion coefficient is 1.5), and the clustering results distinguished between the endemic and common genes, as depicted by the Venn diagram [64, 65].

The Mafft (Table S6) software was subsequently used for multiple sequence comparisons of supergenes [66]. A suitable base substitution model was selected, followed by constructing a species-based maximum likelihood (ML) phylogenetic tree [27, 67, 68]. Moreover, the mcmctree tool of the PAML (Table S6) software package (parameters: burn-in = 5,000,000, sample-number = 1,000,000, sample-frequency = 50) was used to estimate the differentiation time based on the single-copy gene family [69, 70]. The gene families of each species were then analyzed using the Café (Table S6) software. The numbers of gene family contractions and expansions on each evolutionary branch were obtained, and their occurrences were assessed. After the threshold value of the family-wide *P*-value was set at 0.05, GO functional enrichment analysis was performed for genes in these families.

Furthermore, protein-coding sequences were identified using the positive selection approach by distinguishing between synonymous substitutions (Ks) and non-synonymous substitutions (Ka) [71]. The analysis method of the Branch-site model proposed in 2002 can detect the forward selection occurring in a specific evolutionary lineage and affecting only a portion of genome sites [72]. This study used the Branch-site model to detect the forward selection acting on the protein-coding sequence. Briefly, one-to-one orthology proteins from white clover and related species were selected, and homologous protein sequences were compared using the default parameters of PRANK. The alignment results were filtered with Gblocks (parameters: -t = c -e = .ft -b4 = 5 -d = y), and CODEML in PAML was used to test the positive selection in a specific branch, which only affected some loci. Thereafter, the Chi2 program in PAML (Table S6) was used to check and correct multiple hypotheses (Main parameters include; degree of freedom = 2), after which we obtained the positive selection genes.

Ks values for homoeologous loci of the constructed genome were used to detect WGD events [73]. Moreover, Blastp was used to compare the longest protein sequence encoded by the white clover genes. The MCScanX (Table S6) software was subsequently used to filter the comparison results, and the Yn00 tool of the PAML (Table S6) software package was used to calculate the synonymous replacement rate [74, 75]. Furthermore, a density distribution map based on the Ks values of all paralog and ortholog gene pairs between the genomes of white clover, red clover, and other related species was drawn using MATLAB [26, 76]. The gene comparisons were then made between and within related species.

## Supplementary Information

**Additional file 1.**

**Additional file 2.**

**Additional file 3: Table S1.** Benchmarking Universal Single-Copy Orthologs analysis of white clover.

**Additional file 4: Table S2**. Chromosome sequence distribution statistics.

**Additional file 5: Table S3.** Repeat sequences results.

**Additional file 6: Table S4.** GO, eggNOG, NR, KEGG and SP annotation results.

**Additional file 7: Table S5**. The gene families described (including their GO terms) and their numbers between white clover and the gene family expansion in white clover.

**Additional file 8: Table S6.** URLs and code of the software.

## Data Availability

All data generated and analyzed during this current study are available in the Grassland Agri-husbandry Research Center, Qingdao Agricultural University with permission from the Competent Authority. All raw data data were submitted in NCBI Database (SAMN22208873, SAMN33387310, SRR16288262) and the genome assembly and annotation were uploaded in the dedicated public repositories (De novo assembly of Trifolium repens: 10.6084/m9.figshare.23266319, genome annotation of Trifolium repens: 10.6084/m9.figshare.23266532). The details of software used are in Table S6. Biological materials used in this study available from the corresponding author.

## Abbreviations

NT: Nucleotide Sequence Database
PE: Paired-end
NGS: Next-Generation Sequencing
CCS: Circular Consensus Sequencing
BUSCO: Benchmarking Universal Single-Copy Orthologs
Hi-C: High-throughput chromosome conformation capture
MITEs: Miniature inverted repeat transposable elements
LTR: Long terminal repeat
LTR-RT: Long terminal repeat retrotransposons
ncRNA: Non-coding RNA
NR: NR is the NCBI non-redundant protein database
GO: Gene Ontology
KEGG: Kyoto Encyclopedia of Genes and Genomes
WGD: Whole Genome Duplications
BWA: Burrows-Wheeler-Alignment Tool
