# Supplementary figures and images for: High-quality chromosome-level de novo assembly of the Trifolium repens
Source: BMC Genomics. 2023 Jun 13;24:326. doi: 10.1186/s12864-023-09437-8 (PMC10265827; doi:10.1186/s12864-023-09437-8)

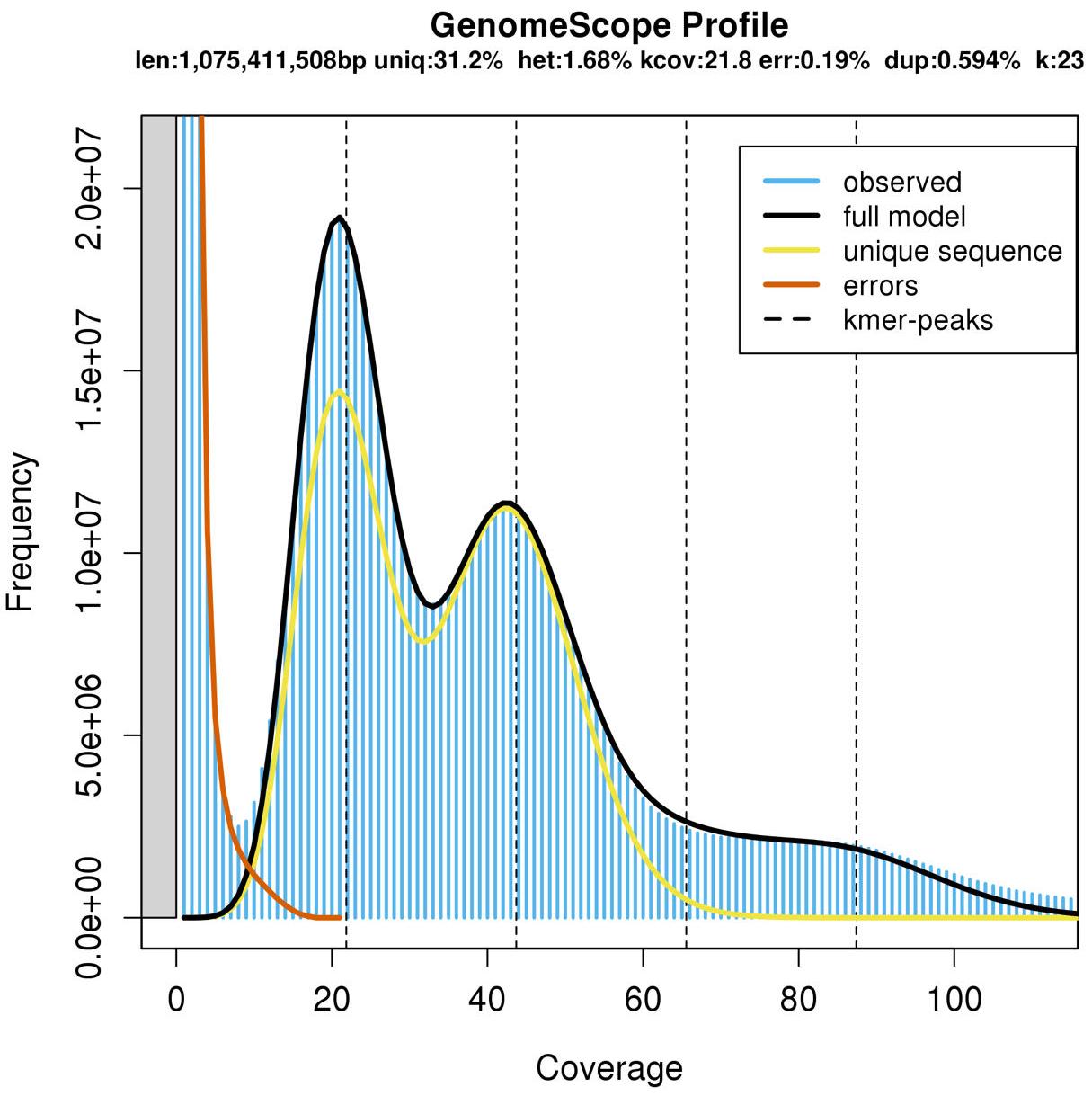

Supplement: Supplementary file 1 — Additional file 1. [file 12864_2023_9437_MOESM1_ESM.jpg]

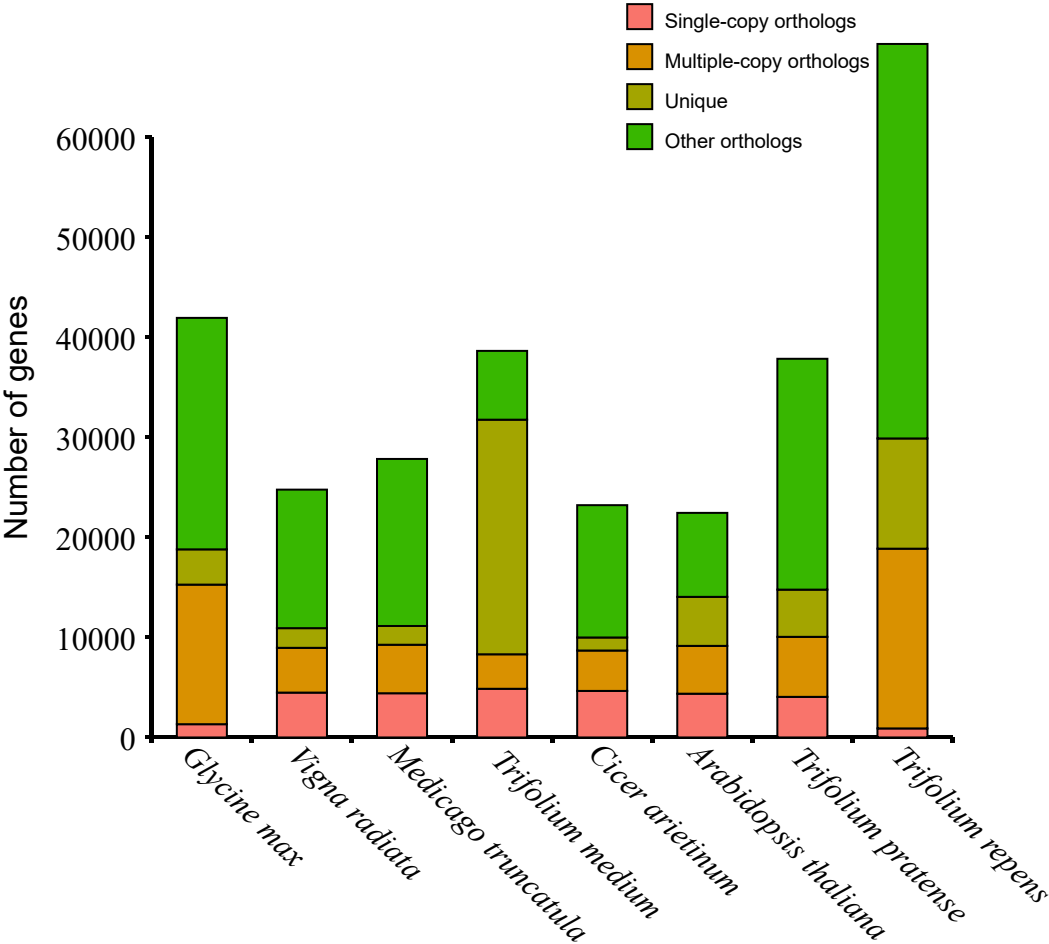

Supplement: Supplementary file 2 — Additional file 2. [file 12864_2023_9437_MOESM2_ESM.pdf]
