# Supplementary material for: High-quality chromosome-level de novo assembly of the Trifolium repens
Source: BMC Genomics. 2023 Jun 13;24:326. doi: 10.1186/s12864-023-09437-8 (PMC10265827; doi:10.1186/s12864-023-09437-8)
Supplement: Supplementary file 3 — Additional file 3: Table S1. Benchmarking Universal Single-Copy Orthologs analysis of white clover. [file 12864_2023_9437_MOESM3_ESM.pdf]

Table S1. Benchmarking Universal Single-Copy Orthologs analysis of white clover

BUSCO notation assessment results	
Complete single-copy BUSCOs	98.5%
Complete and single-copy BUSCOs	16.00%
Complete Duplicated BUSCOs	82.50%
Fragmented BUSCOs	0.10%
Missing BUSCOs	1.40%
Total BUSCO groups searched	2326
