# Supplementary material for: High-quality chromosome-level de novo assembly of the Trifolium repens
Source: BMC Genomics. 2023 Jun 13;24:326. doi: 10.1186/s12864-023-09437-8 (PMC10265827; doi:10.1186/s12864-023-09437-8)
Supplement: Supplementary file 4 — Additional file 4: Table S2. Chromosome sequence distribution statistics. [file 12864_2023_9437_MOESM4_ESM.pdf]

Table S2. Chromosome sequence distribution statistics

ID	Chromosome	Sequence Number	Sequence Length (bp)
1	Chr01	11	81,754,482
2	Chr02	26	72,856,632
3	Chr03	7	70,812,884
4	Chr04	15	69,481,492
5	Chr05	9	66,386,749
6	Chr06	10	65,913,581
7	Chr07	5	64,655,134
8	Chr08	13	64,541,361
9	Chr09	7	64,016,903
10	Chr10	16	62,980,941
11	Chr11	16	62,644,447
12	Chr12	11	62,405,417
13	Chr13	2	61,529,956
14	Chr14	9	59,709,593
15	Chr15	3	56,043,553
16	Chr16	6	55,734,426
	Total	168（40.6%）	1,041,467,551（95.06%）
