# Supplementary material for: High-quality chromosome-level de novo assembly of the Trifolium repens
Source: BMC Genomics. 2023 Jun 13;24:326. doi: 10.1186/s12864-023-09437-8 (PMC10265827; doi:10.1186/s12864-023-09437-8)
Supplement: Supplementary file 5 — Additional file 5: Table S3. Repeat sequences results. [file 12864_2023_9437_MOESM5_ESM.pdf]

Table S3. repeat sequences results

	Repeat type	Classification	Number
Class I: Retrotransposon	LTR-Retrotransposon	Copia	282,245
		Gypsy	160,786
		Others	443,194
	Non-LTR Retrotransposon	LINE	71,845
Class II: DNATransposon	Subclass I	CMC-EnSpm	4,284
		MULE-MuDR	12,493
		PIF-Harbinger	2,823
		hAT-Ac	1,385
		hAT-Tag1	1,768
		Others	560,300
	Subclass II	Helitron	6,987
	Tandem Repeat	Simple repeat	4,092
	Other	-	148
	Unknown	-	471,061
	Total	-	2,023,411

Masked(bp)	Masked(%)
14,589,712	13.56%
125,896,098	11.49%
139,112,843	12.70%
27,400,733	2.50%
1,678,603	0.15%
5,633,452	0.51%
974,162	0.09%
690,599	0.06%
520,015	0.05%
134,900,423	12.31%
2,731,100	0.25%
508,915	0.05%
19,826	0.00%
83,741,318	7.64%
672,397,799	61.37%
