# Supplementary material for: High-quality chromosome-level de novo assembly of the Trifolium repens
Source: BMC Genomics. 2023 Jun 13;24:326. doi: 10.1186/s12864-023-09437-8 (PMC10265827; doi:10.1186/s12864-023-09437-8)
Supplement: Supplementary file 8 — Additional file 8: Table S6. URLs and code of the software. [file 12864_2023_9437_MOESM8_ESM.pdf]

Table S6. URLs and code of the software.

Number	Software	Version	Code	Website
1	Jellyfish	v1.1.11	-m 19 -t 60 -C -c 8	http://www.cbcb.umd.edu/software/jellyfish/
2	Minia	v3.2.4	Default code	https://github.com/GATB/minia/releases
3	Smrtlink	v8.0	Default code	https://www.pacb.com/
4	Hifiasm	v0.15.2	-l 0	https://github.com/chhylp123/hifiasm
5	Purge_dups	v1.2.3	-2 -T changed.cutoffs -l XXX - mXXX -u XXX	https://github.com/dfguan/purge_dups
6	Minimap2	v 2.13	-I6G -ax map-pb -t 60	https://github.com/lh3/minimap2
7	BWA	v0.7.15	mem -t 96 -M -S	http://bio-bwa.sourceforge.net/
8	BUSCO	V4.0.6	-c 60 - m geno --offline	https://busco.ezlab.org/
9	Blast	v2.12.0	-evalue 1e-5	https://blast.ncbi.nlm.nih.gov/Blastcgi
10	JUICER	v1.6.2	Default code	https://github.com/aidenlab/juicer
11	3D-DNA	180922	Default code	https://github.com/theaidenlab/3d-dna
12	Augustus	v3.2.2	--species --alternatives-from-evidence --alternatives-from-sampling --allow_hinted_splicesites=atac	http://augustus.gobics.de/binaries/
13	EVM	r2012-06-25	--gene_predictions --protein_alignments --	https://sourceforge.net/projects/evidencemodeler/
14	GeneMark-ESSuite	v4.57	--ET --cores 48 --max_gap 3000	http://topaz.gatech.edu/GeneMark/license_download.cgi
15	GeMoMa	v1.7.1	Default code	http://www.jstacs.de/index.php/GeMoMa
16	Glimmerhmm	v3.0.4	Default code	http://ccb.jhu.edu/software/glimmerhmm/
17	LTR Finder	v1.07	-D 15000 -d 1000 -L 7000 -l 100 -p 20 -C -M 0.9	https://github.com/xzhub/LTR_Finder
18	LTR retriever	v2.8.2	Default code	https://github.com/oushujun/LTR_retriever
19	PASA	v r20140417	-c alignAssembly.config -C -R --ALIGNERS blat,gmap --CPU 32 --stringent_alignment_overlap 30.0	https://sourceforge.net/projects/pasa/files/stats/timeline
20	MITE Hunter	v1.0	-n 5 -c 20 -S 12345678	http://target.iplantcollaborative.org/mite_hunter
21	RepeatMasker	v4.1.0	-nolow -norna -gff -engine ncbi -parallel 20 -no_is	http://www.repeatmasker.org/RMDownload.html
22	RepeatModeler	v2.0	-engine ncbi -pa 60 -database	http://www.repeatmasker.org/RepeatModeler/
23	SNAP	v6.0	Default code	http://snap.stanford.edu/snap/download.html

24	tRNAscan-SE	v2.0	Default code	http://lowelab.ucsc.edu/tRNAscan-SE/
25	Café	v3.1	-p 0.05 -t 4	https://github.com/hahnlab/CAFÉ
26	Lastz	v1.04	Default code	http://www.bx.psu.edu/miller_lab/dist/README.lastz-1.02.00/
27	Mafft	v7.310	--auto --anysymbol --thread	https://mafft.cbrc.jp/alignment/software/
28	Mcscan	vX	-s {num}	http://chibba.agtec.uga.edu/duplication/mcscan/
29	Mummer	v4.1	-g 1000 -r -q -l 200 -rcloT	https://github.com/mummer4/mummer
30	OrthoMCL	v2.0.9	--abc -I 1.5	https://orthomcl.org/orthomcl/
31	PAML	v4.9e	Default code	http://abacus.gene.ucl.ac.uk/software/paml.html
